# Patient Perceptions of Weight Stigma Experiences in Healthcare: A Qualitative Analysis

**DOI:** 10.1111/hex.70013

**Published:** 2024-09-02

**Authors:** Kathleen M. Robinson, Kimberley A. Robinson, Aaron M. Scherer, Melissa Lehan Mackin

**Affiliations:** ^1^ Department of Internal Medicine University of Iowa Hospitals and Clinics Iowa City Iowa USA; ^2^ Division of Endocrinology University of Iowa Hospitals and Clinics Iowa City Iowa USA; ^3^ Department of Sociology and Psychology Columbia College Sonora California USA; ^4^ Health Science Campus, College of Nursing University of New Mexico College of Nursing Albuquerque New Mexico USA

**Keywords:** attribution theory, inductive, qualitative research, survey, weight stigma, weight stigma in healthcare

## Abstract

**Background:**

Weight stigma is the social devaluation and denigration of individuals because of their excess body weight, resulting in poorer physical and mental health and healthcare avoidance. Attribution Theory and Goffman's theory of spoiled identity provided a general overarching framework for understanding weight stigma experiences.

**Objective:**

Our purpose was to explore weight stigma experiences from a broad range of perspectives emphasizing identities typically excluded in the weight stigma literature.

**Design:**

We conducted a qualitative descriptive study with data drawn from 73 substantive narrative comments from participants who responded to a larger survey.

**Results:**

Analysis developed five themes: *Working on weight*, *Not being overweight*, *Lack of help and empathy*, *Exposure and embarrassment* and *Positive experiences*. Individuals who would be clinically assessed as overweight, especially men, often did not identify with having a weight problem and found the framing of personal responsibility for weight empowering. Participants with larger body sizes more often attributed embarrassment and shame about weight to treatment in the clinical setting. Older participants were more likely to have positive experiences.

**Conclusions:**

The findings suggest ongoing tension between the framing of weight as a personal responsibility as opposed to a multifactorial condition with many uncontrollable aspects. Gender, age and body size shaped respondent perspectives, with some young male respondents finding empowerment through perceived personal control of weight. The healthcare system perpetuates weight stigma through lack of adequate equipment and excessively weight‐centric medical counselling. Recommending a healthy lifestyle to patients without support or personalized medical assessment may perpetuate weight stigma and associated detrimental health outcomes.

**Patient or Public Contribution:**

Patients with obesity and overweight were integral to this study, providing comments for our qualitative analyses.

## 1 Introduction

Weight stigma is defined as the ‘social devaluation and denigration of individuals because of their excess body weight, and [it] can lead to negative attitudes, stereotypes, prejudice, and discrimination’ [1]. Weight stigma is pervasive, and common in healthcare environments [2, 3]. In one study of Weight Watchers (WW) participants in the United States, 41.3% of respondents reported experiencing weight stigma from a physician [3]. Weight stigma can lead to healthcare avoidance, and worsening of mental and physical health, as well as poor weight loss outcomes for treatment‐seeking individuals [4, 5, 6, 7, 8, 9, 10]. Weight stigma constitutes a significant barrier to the receipt of healthcare, a detriment to quality of life, and is fundamentally contrary to the ethical practice of providing healthcare [1, 11, 12].

Previous qualitative studies have explored how experiences of weight stigma shape clinical interactions and lead to avoidance of healthcare [13, 14, 15]. However, most of these studies emphasize the experiences of women, often in the context of seeking medical treatment for obesity. Far less is known about how men, older individuals, those with a lesser degree of excess body weight or nontreatment‐seeking individuals experience weight stigma in healthcare. These are substantial gaps in the literature.

Through this research, we aimed to better understand how a diverse group of respondents experience weight stigma in healthcare. Attribution Theory was a general guiding framework for our analysis of qualitative data in the context of Goffman's theory of stigmatized or ‘spoiled’ identity [16, 17]. Goffman argues that stigma can arise from many statuses, including bodily traits, character defects and chronic illness. As a result of such characteristics, individuals can develop a ‘spoiled identity’, experiencing shame, self‐doubt and lower self‐esteem. He posits that stigmatized individuals engage in attempts to manage impressions of themselves to reduce the likelihood of being discredited. Attribution Theory posits that people make attributions about the cause and controllability of a condition that leads to inferences about responsibility and ultimately influences the care and support that people receive. Attribution Theory has been used previously to explain discrimination and stigmatization in other health conditions such as mental illness, addiction and COVID‐19 [18]. Psychologists identify several attributional dimensions to explain how individuals interpret events, including controllability, stability and internality. Controllability refers to the extent to which an individual has control over their behaviour or trait. Stability refers to how changeable or stable the trait or behaviour is over time. Internality is concerned with whether the cause of the behaviour or trait is viewed as internal to the person or external [19, 20]. In his work, theorist Bernard Weiner examines the stigmatization and causal framing of obesity, and how understandings of causality shape behaviour and attitudes towards people with obesity [17, 21]. He argues that the perception of controllability is the most important factor in promoting rejection and social distancing that people with obesity may experience [19]. Although evidence shows that there is often an underlying biological cause for weight gain, he found that it is most often treated as a controllable condition, contributing to heightened stigma [21]. Moreover, in his work, those who viewed obesity as controllable expressed less pity, greater anger and less desire to help [17]. Many of the themes that we developed exemplify aspects of his work.

We used attribution theory as a lens for understanding how people with overweight or obesity (as characterized by body mass index [BMI]) made sense of their healthcare experiences in the context of weight stigma. To accomplish our aims, we used an open‐ended response item that elicited narrative responses from respondents who were part of a larger survey distributed to a national sample from the United States.

## 2 Materials and Methods

This research is part of a larger study that aimed to examine the relationship of weight stigma in healthcare to healthcare avoidance. One open‐ended item that prompted narrative responses from survey respondents asked, ‘Any other comments regarding weight and your experiences at your hospital/clinic?’ Reports on the development of the larger survey and survey results are in development or published elsewhere [22].

The complete survey consists of 24 items related to different domains of weight stigma experienced in healthcare (general experiences, communication, physical examination and the clinical environment), a 5‐item measure of self‐reported healthcare avoidance and a demographics section. This survey, the Weight Stigma in Healthcare Inventory, was administered using a Qualtrics survey panel for 2 months during the summer of 2021. All respondents were 18 years of age or older, able to understand English and had a BMI of 25 kg/m^2^ or higher (as calculated based on reported height and weight). The study targeted a nationally representative sample with respect to rurality, gender, race/ethnicity and age (File S1). Qualtrics recruited our survey sample from among their compensated survey panels by asking screening questions about demographic features. We asked respondents to report gender and included the options male, female and other/please specify. We asked Qualtrics to recruit 49%–50% female respondents, 49%–50% male respondents and 1%–2% unspecified (participants could select male, female or other/please specify). Although our survey study did not ask respondents to specify cis‐ or transgender status, we did give respondents the opportunity to specify/describe gender. No respondents selected this option, so our respondents are treated as cisgender. However, there is potential for nonresponse bias, as gender‐diverse individuals may not have selected the ‘specify/describe’ option. There were no exclusion criteria. The study protocol was approved by the University of Iowa IRB (# 201910736).

Of the 395 survey respondents, 73 provided substantive responses to the open‐ended item that were subject to analysis. The analysis excluded narratives that were one‐word responses, nonsensical responses or responses that were not related to weight and healthcare experiences. This study was approved by the Institutional Review Board, using a waiver of documentation of consent, meaning that consent was implied through participation in the survey but was not documented. Participants received compensation for completion of the survey, per Qualtrics protocol (amounting to approximately $5.60 per respondent). The open‐ended response item was optional.

### 2.1 Analytic Strategy

Qualitative analysis was completed using thematic analysis as described by Braun and Clarke [23]. This type of analysis was chosen due its theoretically flexible approach and ability to produce a complex account of the data. Transcripts were reviewed for patterns and were coded to allow for preliminary organization of data into themes. Codes were further analysed, sorted into themes, with attempts to theorize broader meaning and significance of the patterns. At this time, the researchers observed the similarity of the themes to components of Attribution Theory and held ongoing reflexive discussions through a recursive process of inductive and deductive coding to ensure that all data were attended to as the analysis was refined. Ultimately, Attribution Theory was used an organizing framework for the presentation of findings.

### 2.2 A Note on BMI

It has been widely acknowledged in the medical and popular literature that BMI, although useful at a population level, is not an accurate indicator of an individual's health or metabolic status [24, 25]. To some degree, weight categories are socially constructed. Although many individuals have excess body fat, and many individuals have health consequences associated with excess body fat, BMI maps onto these states imperfectly. In this study, we use BMI data as a rough marker for how individuals would typically be classified in clinical encounters. Clinical guidelines classify individuals as being overweight when BMI is 25 kg/m^2^ or greater, and recommend further evaluation and/or treatment [26], potentially giving rise to weight stigma. We do not make claims about respondents' health status or body composition or whether or not they should lose weight. The BMI information reported for individual participants in the results section is intended to provide clinical context only.

### 2.3 Positionality of Research Team

Mindful that our identities can influence our approach, the authors wish to provide the readers with the following information about our backgrounds. K.M.R. is a non‐Hispanic White, cisgender physician scientist trained in endocrinology and obesity medicine. As a teenager, she had obesity and experienced weight stigma, but now falls into the ‘normal’ BMI range. Her dissertation in the history of medicine examined the development of the Fat Acceptance Movement. M.L.M. is a non‐Hispanic White, cisgender nurse scientist with an interest in women's health and extensive qualitative research experience. She has been higher weight her whole adult life and frequently experiences weight stigma. She is also an advocate for person‐centred language and interventions and sees weight as only one facet of holistic health and wellness. K.A.R. is a non‐Hispanic, White, cisgender sociologist with expertise in ethnography. She has experienced a range of weight fluctuations over the course of her life, but now but has stabilized at a normative body weight. A.M.S. is a non‐Hispanic, White, cisgender psychologist with expertise in survey design. He is at a normative body weight.

## 3 Results

The majority of the 73 respondents who left substantive comments were non‐Hispanic White individuals, roughly half men and half women, with a mean age of 47.8 and a mean BMI of 31.5 kg/m^2^ (Table 1). From analysis of participants' narratives on weight and health, five global themes were developed regarding attributions of weight stigma: (1) *Working on weight*; (2) *Not being overweight*; (3) *Lack of help and empathy*; (4) *Exposure and embarrassment*; and 5) *Positive experiences* in healthcare.

**Table 1:** Characteristics of respondents leaving substantive comments.

Respondent characteristics (*N* = 73)	Mean (SD) or *n* (%)
Age (years)	47.8 (16.0)
Race/ethnicity	
White, non‐Hispanic	53 (72.6%)
Black, non‐Hispanic	7 (9.6%)
Hispanic	11 (15.1%)
Other	2 (2.7%)
Gender	
Female	40 (54.8%)
Male	33 (45.2%)
Other (please specify)	0 (0%)
BMI (kg/m^2^)	31.5 (5.1)

Theme 1 *Working on weight*

Respondents: 10 female/4 male, age range 23–61 years (mean 40.1) and BMI range 25.1–41.3 kg/m^2^ (mean 30.7).

Respondents mentioned the importance of working on weight and emphasized the effort that they put into managing weight, framing weight as a matter of personal control and responsibility. We identified two sub‐themes. One group espoused having personal responsibility for weight controlled by personal behaviour, framing it as a burden.

I talk to my doctor every visit and I have hypothyroidism and I get it checked every 4 months. I have lost 20 pounds in 3 months and I am constantly paying attention to what I eat.

(female, age 57, BMI 27.4)

That I have to lose weight to be healthy.

(female, age 24, BMI 41.3)

A second group of respondents framed their experiences with weight control as straightforward, dismissing the effort of controlling weight.

Watch what you eat and exercise and you won't be fat.

(male, age 33, BMI 26.6)

Watch how you eat to get the job done.

(male, age 26, BMI 32.2)

Never focused on my weight as an issue. I know it limits my day to day struggles but not letting it slow me down or hurt my feelings.

(female, age 52, BMI 39.1)

Theme 2 *Not being overweight*

Respondents: 5 female/9 male, age range: 21–71 years (mean 47.4) and BMI range: 26.4–41.1 kg/m^2^ (mean 28.6).

Respondents frequently indicated that they were not overweight or indicated they did not have a significant weight problem. This was often cited in the context of why these individuals did not experience weight stigma. We classified these respondents' comments into two sub‐themes.

Within the first sub‐theme, respondents stated or implied they were not overweight or indicated that they were not excessively overweight. Some of these comments were inflected by anger as a result of being asked about weight in our survey.

I'm not overweight.

(male, age 71, BMI 28.4)

It will probably be uncomfortable for [overweight] people to talk about health.

(male, age 23, BMI 26.4)

I'm not a very overweight person but I still feel like weight loss is encouraged for everyone across the board … I still have been suggested to lose weight in a medical setting.

(female, age 21, BMI 26.4)

I'm not over weight. Don't assume that just because I'm 5′7″ and 189 I'm over weight.

Your survey was actually the only time I felt overweight and I've been to numerous doctors in the last year.

(male, age 47, BMI 29.6)

Within the second sub‐theme, respondents made comments about being naturally heavier. Some respondents indicated that they had a different body type and were not overweight.

Some of us are just heavier than others, does not make us a fat person.

(female age 66, BMI 38.1)

I personally don't feel I'm overweight but doctors claim I am. I'm tall and big, not fat.

(male, age 47, BMI 26.8)

BMI is a joke. People who lift weights like me weigh more because of muscle.

(male, age 45, BMI 26.4)

I have always weighed more [because] of my body type, so I have seen shock on many faces that I weigh this much. I was nearly put out of the military [because] of weight. It is difficult to bear being told by a doctor, ‘You're just getting old and fat.’

(female, age 63, BMI 28.3)

I've always been a bigger dude. Used to be very athletic (weighed 185‐200 in college), but gained weight as an adult post‐college.

(male, age 57, BMI 41.1)

Theme 3 *Lack of help and empathy*

Respondents: 10 female/5 male, age range 18–63 years (mean 39.8) and BMI range 25.1–38.0 kg/m^2^ (mean 30.0).

The third major theme was a lack of help and empathy from health professionals. We developed two sub‐themes that describe the perceived reaction of health providers and the lack or ineffectiveness of weight loss advice.

First, some respondents noted inappropriate affect from healthcare professionals, including lack of emotional support.

I wish people would be more sympathetic and make people feel comfortable. We need health care too and I always feel insecure knowing that the issue at hand will be eclipsed by my weight.

(male, age 51, BMI 31.9)

A male doctor at [a Midwestern hospital] was extremely rude and I heard him saying horrible things about me just outside the door.

(female, age 30, BMI 32.1)

I believe some people lack compassion or sympathy based on people's sizes and it needs to be discussed more, especially in the medical field.

(female, age 18, BMI 32.1)

The second sub‐theme was ineffective or inappropriate weight counselling. Respondents noted ineffective medical advice, recommendations that did not reflect their true habits or emphasis on weight rather than the medical concern that they wanted addressed.

I've asked for [medical] assistance in regards to my weight and was put on another antidepressant and they stated it would help me stop eating but I don't eat a lot and I eat fairly healthy.

(female, age 41, BMI 32.4)

My first visit to a new family doctor was not good. My BMI was only slightly over normal and that was ALL she talked about. No matter what issue I brought up it went back to my weight. I did not see her again.

(female, age 51, BMI 27.1)

My parents thought I was depressed—I was in grad school, self‐medicating with alcohol, feeling helpless, loss of enjoyment for a lot of things, etc—so I went to the doctor. The doctor first told me to pray about it, and then to lose weight. That would help with my mental health. [Endorphins] from exercise would be enough to help since I was ‘too young to truly experience depression’ at 23.

(female, age 27, BMI 27.4)

Theme 4 *Exposure and embarrassment*

Respondents: 3 female/3 male, age range 23–67 years (mean 42.3) and BMI range 28.7–45.7 kg/m^2^ (mean 36.6).

Several respondents described a sense of excess exposure and embarrassment related to systemic inequities in medical equipment or shaming discussions of weight.

When I went for my mammogram I was given a very tiny gown, I could not sufficiently cover myself as I sat in the waiting area, the hallway was open to all medical personnel and families of those having procedures, there was only a half wall. I literally hid behind that wall and was in tears by the time I got into the lab, the technician immediately gave me a blanket to cover me and calmed me until I was ready for the exam. I saw no one who could help me until then. I wanted to put my own clothes back on and go home….I am large breasted and need a larger gown. I feel the people who hand these gowns to you should notice what they are doing and check in on you after you have received them.

(female, age 67, BMI 35.1)

Equipment is the biggest issue that I have found.

(male, age 52, BMI 35.9)

I don't like when they talk about my weight in front of others.

(male, age 28, BMI 28.7)

I had a negative experience with an intern at a clinic this spring and I was very hurt and embarrassed by him….

(female, age 50, BMI 45.7)

Theme 5 *Positive experiences*

Respondents: 15 female/13 male, age range 29–79 years (mean 56.3) and BMI range 26.6–43.3 kg/m^2^ (mean 32.5).

Finally, some respondents noted positive experiences with their healthcare providers specifically in relation to weight management. These participants noted sensitive communication strategies and compassionate care.

At my clinic, the nurse weighs me. But no one brought up weight. And I brought up exercise. So I think they did it right, to let me take the lead, making it easier to lose weight this year, with no pressure.

(female, age 58, BMI 27.0)

My doc lets me know I need to change/improve, but does so in a non‐judgmental, compassionate way. It encourages/motivates me to actually follow her advice—and it's starting to work. I've gone from a high of 370 to 320.

(male, age 47, BMI 41.1)

## 4 Discussion

This study investigated how patients with overweight and obesity experience weight stigma in healthcare, including individuals who were men, older, had a lesser degree of overweight and were not explicitly seeking weight management care. The major themes we developed were (1) *Working on weight*; (2) *Not being overweight;* (3) *Lack of help and empathy*; (4) *Exposure and embarrassment*; and (5) *Positive experiences*.

In summary, many respondents indicated that they were working on their weight, with some finding this process effortful and others dismissing or diminishing the work required. Several respondents did not feel that weight or overweight were relevant health issues in their lives. Most of these individuals had BMI between 25 and 30 kg/m^2^, but a few of these respondents had higher BMIs in the 30–40 s. Participants noted that BMI is an inadequate measure of body fat content, and many observed that some individuals have different, naturally heavier body types. Participants who had negative experiences with healthcare providers perceived them to lack empathy and knowledge—failed to listen to what their patients were telling them, misattributed health complaints to weight and gave bad advice. Unhelpful advice included providing weight loss advice when the patient wanted help with a different problem or treating for depression when the patient wanted help with weight. Participants also noted a lack of appropriate equipment and privacy, linking these deficits to embarrassment and humiliation. Finally, participants noted that healthcare providers were most helpful and effective when they were empathetic and approached the topic of weight management in a non‐judgemental manner.

The theme *Working on weight* was reflective of the philosophy of individual responsibility for weight management. One group exemplified a resigned acceptance of needing to lose weight, whereas the other group found the process easy and potentially empowering. Consistent with other studies on weight stigma, in which interviewees focused on a ‘search for dignity’ or a ‘moral duty’ to lose weight, many respondents emphasized the need to pay attention to eating habits, exercise and expressed that they had a weight issue [14, 27, 28, 29, 30]. This perspective countered stereotypes of people with obesity, who have often been characterized as lazy, lacking in self‐discipline and overindulgent [16, 31, 32]. Our participants who endorsed ‘working on weight’ actively countered these stereotypes by emphasizing their extensive efforts to manage weight.

Some aspects of the theme *Working on weight* were strongly gendered. Of those who found ‘working on weight’ difficult, all nine were women. These individuals were trying to address weight proactively with medical professionals as partners in weight management, people who care about their health. In their comments, women emphasized the need and strong desire to lose weight. One woman commented on her persistent, ongoing weight management efforts, and another commented on her work seeking medical help. Although women highlighted their work on losing weight, their comments focused on struggle, effort and the consequences of higher weight.

Of the respondents who dismissed the effort of weight control or found these efforts empowering, there were four men and one woman. They also had lower BMI levels and were younger than the study average. Similar to the respondents we grouped under Theme 2 (‘Not being overweight’), these mostly male respondents may not have perceived their weight as elevated. Additionally, being young may have made them more likely to perceive this success in weight management as easily achieved, given that younger adults tend to weigh less than older adults [33, 34]. For these individuals, working on weight may have felt empowering due to perceived easy success in this domain.

Several of the comments could be interpreted as stigmatizing towards individuals with obesity, such as ‘watch what you eat and exercise and you won't be fat’. In addition to implying that weight management should be easily accomplished by the individual, these comments conversely imply that people at higher body weights are not eating or exercising correctly and that blame lies with them. These comments reflect a lack of sensitivity towards individuals struggling with weight and weight stigma and may hint at gender differences in the propagation of weight stigma linked to the perception of individual responsibility for weight. Although both men and women face approbation for weight [35], men may be more likely to enforce weight‐based gender norms that often punish women for higher weight [36].

Our study differs from others in that a subset of respondents framed working on weight as a straightforward process. In other studies, interviewees espoused ambivalence regarding who should take responsibility for weight management (e.g., providers vs. patients) [13, 14, 15, 29]. This ambivalence was less evident in our study. These respondents described ‘[getting] the job done’ and ‘staying healthy’, indicating a measure of success in their weight management efforts.

Much of the data from the *Working on weight* theme can be productively understood in relation to Attribution Theory. In his work, Weiner found that unstable (reversible) states generally elicited less pity, and were viewed as more controllable [17] and therefore more stigmatized. However, his work focused on the presentation of scenarios to respondents and did not give people with the stigmatized condition the opportunity to counteract or challenge stigma formation. Although potentially facing greater stigma due to healthcare providers viewing obesity as a controllable and unstable condition, some respondents found empowerment through aspiring to lose weight through positive habits. By highlighting what they viewed as the unstable nature of obesity and their commitment to reversing the condition, respondents sought to demonstrate willpower and perseverance. This suggests the possibility that the stigma of unstable health conditions can be mitigated or managed through commitment to future change.

One notable finding was that some respondents with an elevated BMI did not identify as having overweight or obesity (Theme 2: *Not being overweight*). Other studies on weight stigma have often centred on treatment‐seeking populations, and as such, their respondents identified body weight as a medical concern [13, 15, 37]. In contrast, our study elicited comments on weight stigma from adults who may or may not have been seeking treatment for weight. Male respondents with BMIs in the 26–29 kg/m^2^ range were the most likely to be unaware that BMI charts would place them in the ‘overweight’ category. These respondents stated that they did not have overweight, and some expressed anger. Although we attempted to frame our questions in a neutral and inoffensive manner, one respondent expressed anger that he might be characterized as having a high body weight. Another respondent, who was aware of BMI ranges, expressed frustration related to BMI standards. Whether or not these individuals clinically had obesity or overweight, their anger may be an indication of an attempt to distance themselves from a stigmatized identity.

These responses fit with literature showing that men tend to be less aware of BMI and have higher perceived ideal weights as compared to women [38]. In one study, women considered themselves overweight at a BMI of 23.7 kg/m^2^, whereas men considered themselves overweight at a BMI of 26.1 kg/m^2^ [39]. In one study, compared to women, men were less likely to have accurate weight perception (odds ratio [OR] = 0.36), weight dissatisfaction (OR = 0.39) or to have attempted weight loss (OR = 0.55) [40]. Men may prefer heavier body weights and view larger body size as healthier. Men experience lower levels of both weight discrimination and internalized weight bias [41]. Women in the United States are more likely to experience weight stigma and societal norms place more pressure on women to maintain thinness as a means of enacting femininity [36, 42]. This gendered framing may be protective for men with respect to reducing internalized weight bias but may make weight management (when medically indicated) more challenging [43, 44, 45].

A subset of respondents within the theme *Working on weight* emphasized having a naturally heavier body type. These comments were divided equally between men and women, with a trend towards higher BMIs (average 33.6 kg/m^2^). These respondents noted being athletic and exercising, and specifically noted not being ‘fat’. This framing valorizes muscular and athletic bodies, while demonizing fat tissue as a reason for large body size. Conceptualizing fat tissue as morally suspect and unhealthy resonates with deeply held scientific and religious perspectives on adiposity that contribute to weight stigma [46, 47, 48]. By emphasizing their own lack of fatness, participants may have sought to destigmatize larger body size.

From an Attribution Theory perspective, respondents who emphasized having larger body types suggested an uncontrollable, internal cause for higher body weight rather than framing their condition as controllable. Although respondents did not specifically mention genetics, they hinted at an underlying innately heavier phenotype. This framing can be seen as a means to elicit empathy by downplaying the controllability of weight, which could potentially reduce rejection and social distancing. Interestingly, respondents did not point to external causes of weight gain, such as social determinants of health or the lack of availability of healthful foods and exercise opportunities. This demonstrates a general acceptance of the causal framing of obesity as a largely internal concern, similar to what Weiner found in his research [21].

One of our key themes was *Lack of help and empathy* from healthcare providers (Theme 3). Respondents found that healthcare providers offered poorly timed advice when weight was not a top priority for the respondent or healthcare providers overemphasized obesity as a cause for their other health conditions. In some cases, the weight management advice offered was simply unhelpful. Our respondents wanted healthcare providers to strive for the right degree of communication surrounding weight, neither avoiding the topic nor forcing it into every health‐related conversation. Other studies on weight stigma similarly found that weight was overemphasized as a cause of disease, healthcare providers often did not seem to have the necessary training to offer guidance and often viewed weight management treatment with ambivalence [13, 14, 37, 49, 50, 51, 52, 53].

In our study, the lack of essential and appropriate care for weight‐related concerns was associated with a lack of sympathy and empathy. This resonates with other qualitative studies showing that people with obesity often felt that their relationships with physicians were poor. Interviewees have noted patronizing, disrespectful, derogatory comments [13, 52], a sense of less time with physicians and physician emotional withdrawal [15] or less emotional support [54]. Respondents in our study, and these other qualitative investigations, found that their emotional needs in the establishment of a caring, therapeutic relationship, went unmet. Our data point to the possibility that gender may shape expectations of help and empathy. More women than men made comments related to this theme (10 vs. 5) and discussed mistreatment. More women described wanting help, empathy and assistance than weight management as compared to men. Previous studies suggest that women may seek more emotional support around the topic of weight and more patient‐centric communication [55, 56, 57, 58].

Within Theme 4, *Exposure and embarrassment*, respondents expressed feeling physically and emotionally exposed by small gowns, public weighing areas and conversations about weight that could be easily overheard. These experiences were associated with embarrassment and feeling self‐conscious and likely increased tendencies to avoid healthcare. These findings were similar to other studies examining persons with obesity that found medical appointments to be a time of vulnerability and lack of privacy, notably in appointments centred on weight, physical therapy or pregnancy care [49, 50, 52, 53]. Physical vulnerability was exacerbated by lack of equipment designed for larger bodies, including scales, gowns, magnetic resonance imaging (MRI) machines and blood pressure cuffs [52, 59]. In this study and in previous research, interviewees found that degrading attitudes from physicians could be especially detrimental, and conversely, interviewees felt the need for a safe space in which to access medical care [27, 52].

Respondents to Theme 3 were evenly split between men and women (three men, three women), who all reported larger than study average body size. Both men and women may be vulnerable to the negative effects of exposure and embarrassment, often precipitated by lack of appropriate sized equipment or public discussions of weight. Although issues related to communication and responsibility for weight likely impact respondents with a wide range of body size, those with larger body sizes may face additional difficulties with equipment and exposure. Notably, experiences in this category potentially created a great deal of distress, with one woman describing being in tears and another feeling ‘very hurt and embarrassed’.

The themes *Lack of help and empathy* (Theme 3) and *Exposure and embarrassment* (Theme 4) illustrate the consequences of weight stigma when obesity is viewed as entirely controllable. Consistent with Attribution Theory, participant reports reflected a lack of compassion by some providers and an unwillingness to provide help and support for weight loss when weight was viewed as a controllable result of individual choice. This healthcare provider behaviour was also in the context of systems that did not have equipment for larger body sizes or were perceived to use tools that were not relevant to the individual (e.g., BMI), further perpetuating weight stigma and the framing of obesity as a fully controllable attribute demanding personal responsibility. These experiences left individuals unsatisfied with the care that they received and make meeting weight loss goals less likely [60].

Despite the negative experiences shared by persons in this study, some respondents noted *Positive experiences* with healthcare providers (Theme 5). They were able to build strong relationships with clinicians based on sensitive, two‐way communication and often felt empowered to initiate conversations about weight. Multiple other studies have demonstrated the importance of a strong patient–provider relationship, centred on professionalism, compassion, positive two‐way communication and ongoing support [37, 49, 50, 51, 52, 53, 59, 61]. Respondents noted the value of being treated respectfully, compassionately and humanely. Some respondents equated this behaviour with professionalism. Patients with obesity want to be treated as individuals, but also as whole, people situated in unique sociocultural contexts [27, 29, 53, 62].

People making positive comments were evenly split between men and women (13 and 15, respectively), were older than the study average (56.3 years vs. 47.8 years) and had a higher than study average BMI (32.5 kg/m^2^). Several of the men mentioned ‘fairness’, whereas women did not. Otherwise, there were no clearly discernable difference between the positive comments from men and women, with both groups mentioning the importance of respect, compassion and dignity. Given that higher BMI is usually associated with a greater degree of weight stigma [60], we suspect that respondents with positive experiences had higher than study average BMI levels due to confounding with age, as BMI and age are positively correlated [33, 34].

Age may have been the most salient factor in shaping positive experiences with healthcare providers. Other weight stigma studies have found that older individuals may experience less weight stigma in healthcare [41, 63]. This finding has not been extensively explored in the qualitative literature, but we can speculate as to several possible causes. Higher body weight may be less detrimental to health in older individuals, which may in turn lessen weight stigma [64]. Older respondents may have differing body ideals, with weight gain viewed as part of the ageing process and viewed as more socially acceptable [65, 66, 67]. Additionally, older individuals may place greater emphasis on health as opposed to cosmetic concerns [65, 66]. Given that older patients may have more medical comorbidities, clinical conversations may focus more on overall health. Patients may be more likely to view this as holistic, patient‐centred care consistent with the themes discussed above. Finally, there may be generational differences in how older patients view patient–physician interactions [55, 57]. The influence of age on experiences of weight stigma requires further research.

Superficially, the theme of *Positive experiences* (Theme 5) appears to not fit into an Attribution Theory framework; however, when viewed as exemplar experiences, the theme contributes to additional understanding of cause and controllability. Some of our respondents implied that weight was treated as a condition that they could change. Although changeable conditions are sometimes stigmatized, their healthcare providers approached the topic with compassion and a non‐judgemental approach. These healthcare providers may have responded favourably to their patients' efforts at weight control, meaning that although weight was treated as an unstable (more stigmatized) condition, it still might elicit empathy if change was underway. In cases where weight was treated as a predominantly uncontrollable health issue aside from personal responsibility, one that requires management like other health conditions (e.g., heart dysfunction, cancer), individuals felt less stigmatized and more in partnership with their providers. The positive experiences and relationships set the conditions for improved success with weight management.

Our study has several strengths. We included more men and individuals at lower BMIs than previous studies. This strategy brought to light the complexity of weight stigma and weight experiences along the BMI spectrum and in relation to gender, and our analysis exemplifies how Attribution Theory can be a productive framework for understanding weight stigma in clinical settings.

Our study had several limitations. Although the breadth of our sample allowed for unique analyses, we did not use purposive sampling as is usually done in qualitative research. This may have limited our ability to truly drill down into one specific phenomenon within experiences of weight stigma. Our open‐ended prompt asked about ‘other’ experiences in clinic, potentially allowing for comments unrelated to weight. However, in practice, the vast majority of responses emphasized weight and we chose to retain only these comments for our analysis. Differences in attitude towards obesity and overweight likely resulted from our selection of a sample that included more men and inclusion of individuals with lower BMIs than previous studies of people with obesity [13, 14]. Although we were able to look at self‐identified male versus female perspectives, we were not able to include an analysis based on cis/transgender identity. Transgender individuals may not have responded to our survey, given our gender response categories, so these important perspectives are likely under‐represented. In a future data collection, we plan to include SOGI questions to improve inclusivity. Although we requested a nationally representative, diverse sample with respect to race/ethnicity, ultimately, our sample was predominantly non‐Hispanic White. Experiences of weight stigma may be intersectional with other forms of stigma. We hope to further explore this dynamic in future research, utilizing targeted recruitment methods to enhance sample diversity.

## 5 Conclusion

Similar to Attribution Theory, many of our respondents found that framing obesity as a controllable condition for which they were personally responsible increased stigma. As a corollary, emphasizing non‐modifiable contributions to obesity, such as genetics, underlying medical conditions and the social determinants of health, can potentially reduce weight stigma [68]. For our respondents who described being naturally heavier, pointing to an underlying, uncontrollable phenotype may have reduced the personal burden of stigma.

Yet, some of our respondents found the emphasis on personal habits and lifestyle, which can also contribute to obesity, empowering [69]. While accepting the predominant view that obesity is controllable and unstable over time, these respondents suggested that by positively engaging in a healthy lifestyle, they could achieve or maintain their desired weight. Although Attribution Theory is a useful framework for understanding clinicians' views of weight, these perspectives may be malleable based on patients' expressed intentions to work on weight. Young men who see themselves as successful in weight management may be more likely to endorse this framework as empowering.

In addition to a fit with Attribution Theory, our findings illuminate gendered experiences with weight stigma, inflected by age and body size. Women were more likely to describe the work that goes into managing weight, whereas men tended to frame this process as more straightforward. Men were more likely to be unaware of BMI standards or angered by them, whereas both men and women sometimes described their bodies as naturally heavier but not ‘fat’. Women described a concerning lack of help and empathy in medical care more often than men, but both men and women found exposure and lack of equipment deeply problematic. Finally, equal numbers of men and women seemed to have positive experiences with healthcare providers.

Older individuals may experience less weight stigma in healthcare, but less is known about the experiences of this population. More work should be done to determine whether interactions between older patients and their healthcare providers can serve as a model for weight stigma reduction in other populations.

Our research supports several suggestions for clinical practice. First, healthcare providers should work to establish caring and sympathetic relationships with their patients. This therapeutic relationship is the foundation of medical practice. In keeping with Attribution Theory, healthcare providers should reflect on their own understandings of body weight, recognizing that overemphasizing the controllability of obesity may be associated with increased blaming and less empathetic reactions to patients [17]. Healthcare providers should encourage patients to make healthy lifestyle choices but should acknowledge that obesity is a chronic medical condition that is not entirely controllable, and leading a healthy lifestyle in and of itself is rarely enough to cure obesity [70].

The privacy of patients should be respected. In medical contexts, patients often feel exposed and vulnerable. Proper equipment and gowns should be available to support better medical care. Privacy should also be respected by discussing weight in a manner that is discreet and professional. Patients should be assessed and treated holistically. Rather than offering blanket advice, recommendations related to weight should take into consideration individual needs and the context of care. BMI can be used as a screening tool but further evaluation is required [12, 25]. Weight should generally not be addressed at visits related to acute health concerns unless directly related. Yet, the topic should also not be ignored [53]. One of the key ways to accomplish this balance is to use the 5As model of counselling, which entails asking permission to discuss weight [71, 72].

Healthcare providers should be aware that not all patients with higher BMI have weight‐related health conditions or perceive higher weight as a health concern. Men, and individuals with a BMI just over 25 kg/m^2^, may be less likely to consider weight as a health concern. These individuals require careful evaluation to determine if they do indeed have a less medically concerning phenotype, such as higher muscle mass, a gynoid body fat distribution or good metabolic health [68, 73].

Unfortunately, patients themselves often express weight bias directed towards themselves or others. Healthcare providers in this scenario might remind patients that higher levels of body fat are not always unhealthy, obesity is a complex condition with a strong genetic component and recommend self‐compassion.

In conclusion, weight stigma is a type of discrimination in healthcare that is highly prevalent and deeply distressing to patients and interferes in meaningful patient–provider relationships and intervention outcomes. Healthcare providers should make every effort to establish strong relationships with patients; understand the complex aetiologies of obesity and overweight beyond personal responsibility; address weight as part of holistic evaluation; and provide compassionate care in an environment that feels safe.

## Author Contributions

**Kathleen M. Robinson:** conceptualization, writing–original draft, formal analysis, methodology. **Kimberley A. Robinson:** writing–review and editing, formal analysis. **Aaron M. Scherer:** conceptualization, writing–review and editing, supervision. **Melissa Lehan Mackin:** conceptualization, writing–review and editing, methodology, formal analysis, supervision.

## Conflict of Interest

The authors declare no conflict of interest.

## Supporting information

Supporting information.

## Data Availability

Data may be available upon request.
