# Supplementary material for: Patient Perceptions of Weight Stigma Experiences in Healthcare: A Qualitative Analysis
Source: Health Expect. 2024 Sep 2;27(5):e70013. doi: 10.1111/hex.70013 (PMC11369018; doi:10.1111/hex.70013)
Supplement: Supplementary file 1 — Supporting information. [file HEX-27-e70013-s001.docx]

Supplement 1. Demographics of Sample Requested from Qualtrics ®

| Characteristic | | Percentage (Approximate) |
| --- | --- | --- |
| Gender |  |  |
|  | Male | 49-50% |
|  | Female | 49-50% |
|  | Unspecified | 1-2% |
| Age (years) |  |  |
|  | 18-34 | 33% |
|  | 35-54 | 33% |
|  | 55+ | 33% |
| Race/ Ethnicity |  |  |
|  | Non-Hispanic White | 63% |
|  | Non-Hispanic Black | 12% |
|  | Hispanic | 17% |
|  | Other | 8% |
| Rurality |  |  |
|  | Rural | 20-30% |
|  | Urban | 30-40% |
|  | Suburban | 30-50% |

Demographic targets were met by asking screening questions about gender, age, race/ethnicity, and self-reported urban/ rural / suburban status prior to survey initiation.
